# Diurnal Transcriptome and Gene Network Represented through Sparse Modeling in *Brachypodium distachyon*

**DOI:** 10.3389/fpls.2017.02055

**Published:** 2017-11-28

**Authors:** Satoru Koda, Yoshihiko Onda, Hidetoshi Matsui, Kotaro Takahagi, Yukiko Uehara-Yamaguchi, Minami Shimizu, Komaki Inoue, Takuhiro Yoshida, Tetsuya Sakurai, Hiroshi Honda, Shinto Eguchi, Ryuei Nishii, Keiichi Mochida

**Affiliations:** ^1^Graduate School of Mathematics, Kyushu University, Fukuoka, Japan; ^2^Cellulose Production Research Team, Biomass Engineering Research Division, RIKEN Center for Sustainable Resource Science, Kanagawa, Japan; ^3^Faculty of Data Science, Shiga University, Shiga, Japan; ^4^Kihara Institute for Biological Research, Yokohama City University, Kanagawa, Japan; ^5^Integrated Genome Informatics Research Unit, RIKEN Center for Sustainable Resource Science, Kanagawa, Japan; ^6^Research and Education Faculty, Multidisciplinary Science Cluster, Interdisciplinary Science Unit, Kochi University, Kochi, Japan; ^7^The Institute of Statistical Mathematics, Tokyo, Japan; ^8^Institute of Mathematics for Industry, Kyushu University, Fukuoka, Japan; ^9^Institute of Plant Science and Resources, Okayama University, Okayama, Japan

**Keywords:** *Brachypodium distachyon*, transcriptome, autoregressive with exogenous variables (ARX) model, group-SCAD, gene network inference

## Abstract

We report the comprehensive identification of periodic genes and their network inference, based on a gene co-expression analysis and an Auto-Regressive eXogenous (ARX) model with a group smoothly clipped absolute deviation (SCAD) method using a time-series transcriptome dataset in a model grass, *Brachypodium distachyon*. To reveal the diurnal changes in the transcriptome in *B. distachyon*, we performed RNA-seq analysis of its leaves sampled through a diurnal cycle of over 48 h at 4 h intervals using three biological replications, and identified 3,621 periodic genes through our wavelet analysis. The expression data are feasible to infer network sparsity based on ARX models. We found that genes involved in biological processes such as transcriptional regulation, protein degradation, and post-transcriptional modification and photosynthesis are significantly enriched in the periodic genes, suggesting that these processes might be regulated by circadian rhythm in *B. distachyon*. On the basis of the time-series expression patterns of the periodic genes, we constructed a chronological gene co-expression network and identified putative transcription factors encoding genes that might be involved in the time-specific regulatory transcriptional network. Moreover, we inferred a transcriptional network composed of the periodic genes in *B. distachyon*, aiming to identify genes associated with other genes through variable selection by grouping time points for each gene. Based on the ARX model with the group SCAD regularization using our time-series expression datasets of the periodic genes, we constructed gene networks and found that the networks represent typical scale-free structure. Our findings demonstrate that the diurnal changes in the transcriptome in *B. distachyon* leaves have a sparse network structure, demonstrating the spatiotemporal gene regulatory network over the cyclic phase transitions in *B. distachyon* diurnal growth.

## Introduction

Gene regulatory networks (GRNs) represent computationally inferred and experimentally verified molecular relations between genes, which facilitates understanding of gene functions in the context of biological processes (Barabasi and Oltvai, 2004; Davidson and Levin, 2005; Emmert-Streib et al., 2014a). With high-throughput data acquisitions in omics analyses (Fukushima et al., 2009; Moreno-Risueno et al., 2010), GRNs have been applied in a broad range of biological interests. They have been used to identify interactions of genes involved in particular biological processes, such as development (Pattanaik et al., 2014), physiological responses (Urano et al., 2010), metabolism (Tohge and Fernie, 2012), diseases (Emmert-Streib et al., 2014b), and evolution (Erwin and Davidson, 2009).

Being sessile, higher plants have evolved physiological systems to rapidly respond and adapt to environmental changes through alteration of their gene expression states (López-Maury et al., 2009; Hirayama and Shinozaki, 2010; Scheres and van der Putten, 2017). To date, well-studied GRNs of some biological systems have been reported in plants (Krouk et al., 2013), such as auxin signaling (Sankar et al., 2011; Havens et al., 2012; Vernoux et al., 2014), circadian clock (Salazar et al., 2009; Akman et al., 2012; Pokhilko et al., 2012), and flower development (Espinosa-Soto, 2004; La Rota et al., 2011; Jaeger et al., 2013), mainly based on available information regarding the molecular bases of these biological functions in the model plant *Arabidopsis thaliana* (Krouk et al., 2013). Despite methodological advances in molecular biology to experimentally identify gene and/or protein interactions (Reece-Hoyes et al., 2011; Braun et al., 2013; Boeva, 2016), inferring GRNs based on high-throughput biological datasets has been a long-standing challenge in computational biology in terms of throughput, comprehensiveness, and cost-efficiency in various plant species.

Various algorithms for the inference of relations between genes from high-throughput gene expression data have been proposed to decode gene networks. Although gene co-expression networks (GCNs) do not provide dependencies estimated between genes, they have been widely applied in various species using a series of large-scale transcriptome datasets. Specifically, to assess co-expressed gene pairs, mutual information and Pearson’s correlation for predictions of non-linear and linear relations among genes, respectively, have been used in a wide range of species (Steuer et al., 2002; Provart, 2012; Lachmann et al., 2016; Tzfadia et al., 2016). To date, GCNs have been applied to various analyses, such as prioritization of candidate genes, identification of functional modules, and prediction of regulatory factors (van Dam et al., 2017). To infer GRNs from gene expression data, which represent possible causalities between genes, various computational methods have been proposed (Lee and Tzou, 2009). For example, Bayesian inference and state-space modeling have been applied using time-series gene expression datasets to estimate influences along with chronological state transitions (Lèbre, 2009; Wu et al., 2011). Through a visualization of gene relations, GRNs enable us to identify possible gene–gene relationships and hub genes that possibly regulate many subordinated genes and participate in various biological processes (Karlebach and Shamir, 2008; Thompson et al., 2015).

A kind of state-space modeling, the autoregressive with exogenous variables (ARX) model, has been used to identify relations between genes based on their time-series data, including gene expression profiles (Michailidis and D’Alche-Buc, 2013). Owing to the sparseness of GRNs, statistical sparse estimation methods such as Lasso and Elastic net have been applied to GRN estimation, and these results have suggested their feasible functions for their network inference (Fujita et al., 2007; Shimamura et al., 2009; Shojaie and Michailidis, 2011). Among such sparse regularization approaches, smoothly clipped absolute deviation (SCAD) specifically provides an oracle property, which is a desired asymptotic property for sparse estimation (Fan and Li, 2001). Thus, it is expected that the oracle property performs significant roles in variable selection processes as compared with the conventional methods, such as Lasso and Elastic net. For example, an advantage of SCAD is that it estimates possible regulatory functions of genes in a GRN to explore those encoding transcription factors from a time-series transcriptome dataset (Wang et al., 2007).

*Brachypodium distachyon* is a small grass of the subfamily Pooideae, which emerged as a model species for temperate grasses, biofuel crops, and cool-season cereals such as wheat, barley, rye, and oats (Brkljacic et al., 2011; Mochida and Shinozaki, 2013; Kellogg, 2015; Vogel, 2016). Owing to its tractable features, such as small size, simple growth requirements, self-fertility, short life cycle, high-efficiency of *Agrobacterium*-mediated transformation, and small diploid genome size, this species is an ideal system for exploration of genes involved in important traits in grass species. Despite the rapid growth of transcriptome datasets from *B. distachyon* that are useful for constructing GCNs (Sibout et al., 2017), they have not been exploited to infer GRNs that determine relations of dependencies among genes. Thus, in addition to constructing a GCN, it will help infer possible regulatory relations among genes for a deeper understanding of cellular systems in *B. distachyon*.

In the present study, we inferred the gene network of *B. distachyon* based on its diurnal transcriptome dataset. To identify genes that show cyclic expression patterns in the *B. distachyon* transcriptome, we analyzed a series of diurnal transcriptome data of *Brachypodium* leaves and identified its periodic genes. With the expression data of the periodic genes, we first constructed a GCN of the periodic genes. Thereafter, we computed possible dependencies between the periodic genes based on the group SCAD algorithm for sparse modeling to infer GRNs. Moreover, we identified several hub genes highly connected with subordinate genes, and discussed the estimated gene networks in the diurnal transcriptome of *B. distachyon*.

## Materials and Methods

### Plant Materials

The accession Bd21 of *B. distachyon* (W6 36678), which is a single seed-derived inbred line from accession PI 254867, was provided by the National Plant Germplasm System of USDA-ARS. Dry seeds were incubated on wet filter paper in a petri dish at 4°C in the dark for 3 days to synchronize germination. The germinated seeds were grown in a growth chamber at 25°C under a 16 h day photoperiod (60 μmol⋅m^-2^⋅s^-1^) for 4 days. The plants were transplanted in pots filled with autoclaved Pro-Mix BX mycorrhizae (Premier Tech, Rivière-du-Loup, QC, Canada). The potted plants were grown in a growth chamber at 22°C under a 20 h day photoperiod (100 μmol⋅m^-2^⋅s^-1^). The plants were watered with 5,000-fold diluted Professional Hyponex 10-30-20 (Hyponex Japan, Osaka, Japan) every 2 or 3 days.

### Transcriptome Analysis

We sampled leaves from *B. distachyon* Bd21 plants grown in a growth chamber for 19–20 days after synchronized germination for our transcriptome analysis based on RNA-seq using three biological replicates per sample. Total RNA was extracted from the leaves using the RNeasy Plant Mini Kit (Qiagen K.K. Japan, Tokyo, Japan) according to manufacturer’s instructions. The RNA samples were assessed using an Agilent 2100 Bioanalyzer (Agilent Technologies Japan, Ltd., Tokyo, Japan). Libraries for stranded-RNA sequencing were constructed using a TruSeq Stranded mRNA Sample Preparation Kit (Illumina K.K., Tokyo, Japan) according to the manufacturer’s instructions (TruSeq Stranded mRNA Sample Preparation Guide Rev. E; Illumina K.K., Tokyo, Japan). The libraries were assessed using an Agilent 2100 Bioanalyzer (Agilent Technologies Japan). Clonal clusters of the libraries were generated using cBot with a TruSeq Rapid PE Cluster Kit (Illumina K.K.) and sequenced using a Hiseq2500 sequencer with a TruSeq Rapid SBS Kit paired-end sequencing method to obtain 100-bp sequences. The FASTQ file of the sequence data was generated using HCS v2.0.12, Real time analysis v1.17.21.3, and Consensus Assessment of Sequence and Variation v1.8.2 (Illumina K.K.). Library preparation and sequencing were conducted by the Takara Bio Dragon Genomic Center (Takara Bio, Yokkaichi, Japan). The FASTQ files of the raw sequencing reads were submitted to the DDBJ Sequence Read Archive under accession number DRA006158.

### Functional Annotation

Gene Ontology terms for the *B. distachyon* genes were used from the gene annotation information downloaded from Phytozome^1^ (Bdistachyon_314_v3.1.annotation_info). Additional GO terms were associated with the *B. distachyon* genes using GO terms that related to transcripts for *A. thaliana* and rice in the “Best-hit-arabi-name” and “Best-hit-rice-name” row in the annotation file. The GO terms for *A. thaliana*^2^ and rice^3^ were used from the gene annotation information downloaded from Phytozome (Athaliana_167_TAIR10.annotation_info.txt and Osativa_204_v7.0.annotation_info). To reduce bias, GO terms that were assigned to more than 3,000 *Brachypodium* genes were excluded. The dataset of *B. distachyon* genes encoding putative transcription factors was retrieved from the PlanTFDB website^4^ (v4.0, JGI v3.1).

The closest homologs of the *B. distachyon* genes in rice and sorghum were identified by a BLASTP search queried with the deduced protein sequences of *B. distachyon* genes against those of sorghum (annotated in a genome annotation of Phytozome 2.0) and rice (annotated in a genome annotation of MSU ver. 7.0) with a threshold of *e*-value less than 1e-5.

### Gene Expression Analysis

The raw RNA-Seq reads were trimmed using Trimmomatic v0.32 (Bolger et al., 2014) with the LEADING:20 TRAILING:20 SLIDINGWINDOW:4:15 MINLEN:36 commands. The trimmed reads were mapped to the *B. distachyon* Bd21 genome sequence retrieved from the Phytozome website^5^ (Bdistachyon_314_v3.0). The *B. distachyon* RNA-seq reads were mapped to the *B. distachyon* Bd21 genome sequence retrieved from the Phytozome website (Bdistachyon_314_v3.0) using the tmap (v.3.1.4) program using the default parameter settings. Read counts were computed in each annotated gene in the Bd21 genome using the featureCounts program^6^, and based on the reads counts, reads per million mapped reads (RPM) values were calculated. The genes showing RPM value ≥1 in all the three biological replicates in at least one sampling time were defined as expressed genes. The differentially expressed genes (DEGs) were identified using the DESeq2 program (v.1.10.1) (Love et al., 2014) in R v.3.2.4 (R Development Core Team, 2005), with a threshold of *q*-value < 1e-5 (computed using the Benjamini–Hochberg procedure) to assess the deviation of gene expression between 2 days for the sampling.

### Identification of Periodically Expressed Genes

To evaluate the periodicity of the gene expression patterns, we used the wavelet transformation techniques, which are implemented into the WaveletComp package in R^7^. On the basis of *p*-values for significance of periodic power spectra computed for the *B. distachyon* diurnal transcriptome dataset, genes with highly significant power (*p* < 0.01) of the 24 h cycle were selected as candidates for periodically expressed genes in the diurnal transcriptome. In addition, using randomization selection for 6 series of transcriptome datasets for 24 h and 30 permutations of the selected datasets, genes with significant periodicity (*p* < 0.01) for all 30 permutations were considered periodic genes in the diurnal transcriptome. To verify corresponding relations between the putative periodically expressed genes identified by wavelet analysis and sampling time, we carried out a correspondence analysis using the CA package in R.

### Discovery of *Cis*-Regulatory Motifs in Promoter Regions of Periodically Expressed Genes

To discover *cis*-regulatory motifs located in the promoter regions of each periodically expressed gene found in *B. distachyon*, previously reported *cis*-motifs regulating circadian gene expression (Adams and Carré, 2011) were used as queries to search against –2 kb upstream sequences from the putative transcription start site of the periodically expressed gene. To compare the *cis*-regulatory motifs discovered in the promoter regions with other sequenced Pooideae species, the *cis*-motifs were also searched against –2 kb upstream sequences from the putative transcription start site of the closest homologs in rice and sorghum.

### GRN Estimation Based on ARX Models with Group SCAD

We utilized ARX models with group SCAD to estimate possible dependencies of genes in our *B. distachyon* diurnal transcriptome dataset. We performed a regression analysis of the expression patterns of a gene with those of all genes in time order lags 1, 2, and 3 to assess all their possible dependencies corresponding to three models: ARX(1), ARX(2), and ARX(3), respectively. To estimate gene-to-gene relations without any dependencies of specific time lags, we adopted the group SCAD penalty as the regularization term, which enabled us to carry out group estimation. In the ARX(2) model, for instance, each gene gives two explanatory variables: 1- and 2-order delayed variables, in which we assume that the two variables belong to the same group. To cope with observation noise and insufficient data points of the dataset, we applied a bootstrap-like random sampling approach, which generated a set of multiple data matrices while retaining its original temporal structure. Thereafter, we estimated GRNs for each of the data matrices, and assessed confidence for every edge based on the multiple GRNs (Appendices 1 and 2).

## Results and Discussion

### Diurnal Transcriptome of *B. distachyon* Leaves

**FIGURE 1:**
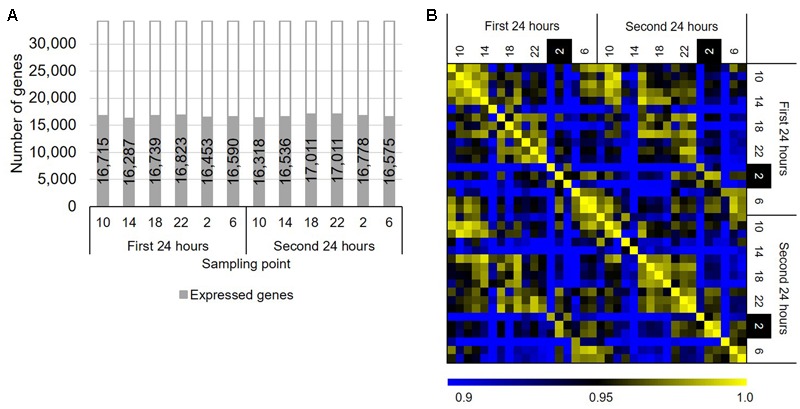
Overview of the diurnal transcriptome in *Brachypodium distachyon* leaves. **(A)** Number of genes significantly expressed (average RPM ≥ 1 in three biological replicates) in the diurnal transcriptome samples. **(B)** Correlation of the gene expression patterns obtained from the transcriptome datasets based on RPM values of 18,578 significantly expressed genes.

To identify genes that show rhythmic expression patterns in *B. distachyon*, we sequenced RNAs from the leaves of *B. distachyon* Bd21 sampled through a diurnal cycle over 48 h. We sampled leaves of *B. distachyon* Bd21 grown under a 20 h light/4 h dark diurnal cycle over 48 h periods at 4 h intervals, a “long day” condition. Although the photoperiod condition did not deviate from the natural conditions, the condition has been used in various studies as a standard condition for *B. distachyon* Bd21 in a growth chamber (Vogel and Bragg, 2009; Rancour et al., 2015; An et al., 2016; Bredow et al., 2016). Transcriptome sequencing revealed that more than 99% of the total reads in each sample were uniquely mapped reads (Supplementary Table S1). We found that 16,287–17,011 genes are significantly expressed, supported by RPM values ≥1 of all three replicated samples at each time point (**Figure 1A**). Moreover, we verified that the gene expression patterns between the series of the first and second days were highly correlated, and the numbers of DEGs between the same time points of those two days were as low as 0.03% (i.e., six genes per 18,578 expressed genes across six time periods between 2 days; **Figure 1B**). This finding suggested that we sequenced the transcriptomes of similar physiological states of the growth stage in *B. distachyon* Bd21 over the 48 h periods. To examine gene expression changes through the diurnal samples, we compared gene expression patterns between all combinations across the six sampling time. As a result, we identified 5,520 DEGs with at least one sample combination, 47.4% of which are found between the samples from 2 to 6 o’clock, suggesting that about half of the DEGs alter their expression pattern from night to morning in response to light signaling in *B. distachyon* (**Supplementary Figure S1**). Although 389 RNA-seq-based transcriptome datasets from *B. distachyon* are present in the public domain (NCBI SRA, as of September 4, 2017), our RNA-seq dataset is the first time-series dataset of a high-resolution diurnal transcriptome in the vegetative growth stage of *B. distachyon*. Therefore, the data will be valuable for understanding the physiological response through diurnal growth in the model grass. In the present study, we applied a 20 h light/4 h dark diurnal cycle, which is the most widely used growth condition for the short life cycle of *B. distachyon* under controlled conditions. It is expected that further analyses with diurnal transcriptome data from different day–night length conditions and/or different growth stages and tissues will facilitate a deeper understanding of molecular systems interacting with diurnal environmental perturbations in temperate grasses.

### Identification of Periodic Genes in *B. distachyon*

**FIGURE 2:**
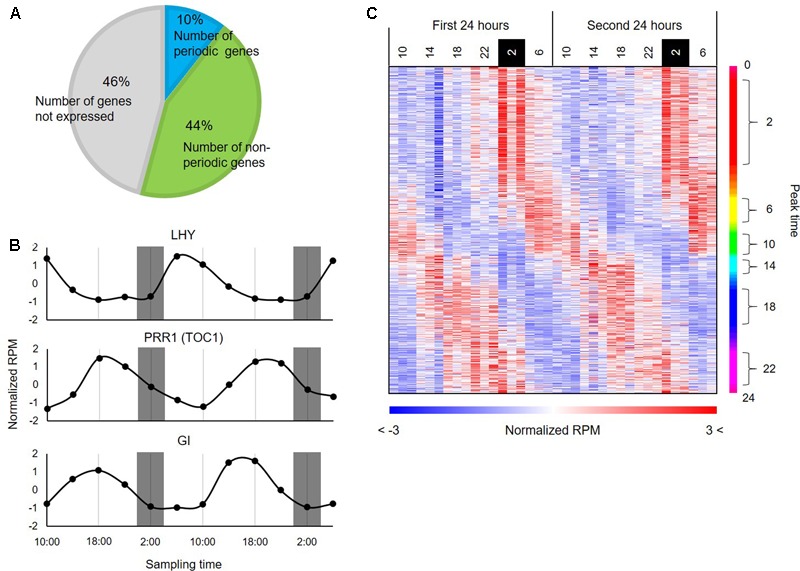
Periodic genes identified from the diurnal transcriptome data of *B. distachyon* leaves. **(A)** Distribution of the periodic genes expressed in *B. distachyon* leaves. The pie chart represents percentages of the periodic genes and non-periodic genes identified through our wavelet analysis. **(B)** Expression patterns of homologs of clock-related genes annotated in rice. **(C)** Expression patterns of the 3,621 periodic genes found in the *B. distachyon* transcriptome. The heatmap represents expression patterns of genes based on the normalized RPM values with a color gradient from blue to red. The genes are vertically sorted along with their estimated peak time.

To infer transcriptional relationships between genes, we first obtained a comprehensive list of genes whose expression patterns are either positively or negatively correlated in the diurnal transcriptome in *B. distachyon*. Using wavelet analysis, we found that 3,621 genes (i.e., 10% of the protein-coding genes annotated in the *B. distachyon* Bd21 genome and 19.5% of expressed genes (i.e., 3621 genes per 18,578 expressed genes) in the series of RNA-seq data (RPM ≥ 1)) showed a rhythmic expression pattern through the diurnal transcriptome datasets (**Figure 2A**). To examine the adequacy of our wavelet screening, we verified the expression patterns of the homologs of genes that are involved in the circadian clock in plants such as rice (Matsuzaki et al., 2015) (**Figure 2B**). Through a correspondence analysis with the 3,621 periodically expressed genes, we also verified that these genes show corresponding relations with the time (**Supplementary Figure S2**). The heatmap of expression patterns for the periodic genes clearly represented a diagonal striped pattern (**Figure 2C**), which is a typical representation of expression patterns of periodic genes, as reported in previous studies on model species such as *Arabidopsis* (Covington et al., 2008) and tomato (Higashi et al., 2016). These results indicate that we successfully identified the periodic genes through wavelet analysis with the diurnal transcriptome dataset in *B. distachyon*. The plant circadian clock plays a crucial role in photosynthesis and regulates carbohydrate and nitrogen metabolism (Adams and Carré, 2011). Moreover, it is involved in physiological responses to various environmental stresses in plants (Grundy et al., 2015). Therefore, the comprehensive list of rhythmically expressed genes will provide essential clues for understanding many circadian rhythm-regulated aspects of growth and development in the model grass. Our comprehensive list of the rhythmically expressed genes and their promoter sequences from *Brachypodium* is expected to compare *cis*-motifs with those from the other sequenced grass species across subfamilies such as rice and sorghum (Supplementary Table S2), which may allow us to understand diversification of GRNs involved in photoperiod responses evolved in Pooideae. Moreover, through comparative analyses with temperate grass crops such as wheat, barley, rye, and oats, functional analyses of the periodic genes in *Brachypodium* may suggest useful strategies for improving the productivity of those crops by modification of the photoperiodic response (Nakamichi, 2015).

### GCN for Periodic Genes in *B. distachyon*

**FIGURE 3:**
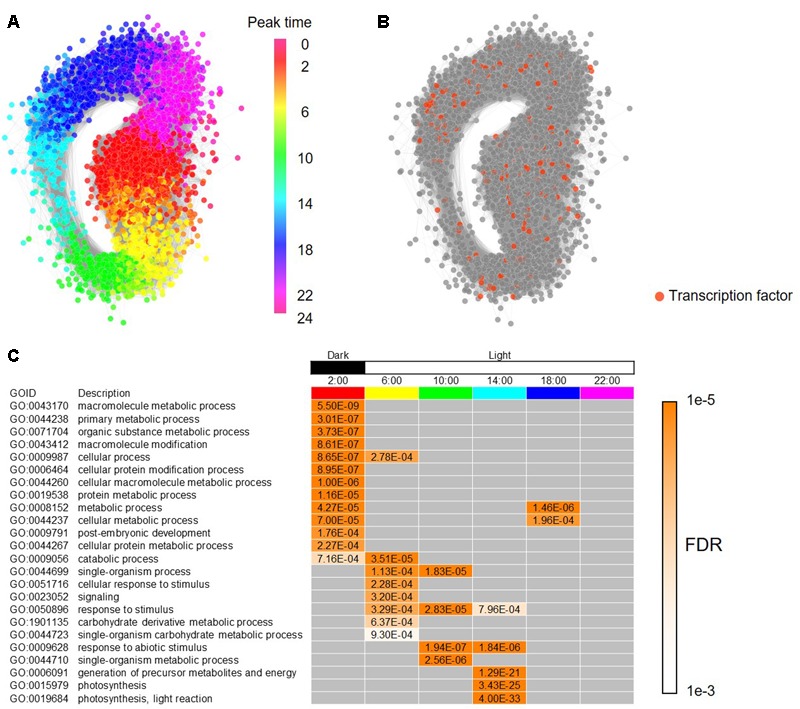
Co-expressed gene network of the diurnal transcriptome in *B. distachyon*. **(A)** A circular co-expressed gene network with nodes of the rhythmic genes colored according to their peak time in their diurnal expressions. **(B)** Genes encoding putative transcription factors found among the rhythmically expressed genes in *B. distachyon*. **(C)** Enriched biological functions in each time-period based on the process ontology.

To build a framework of a regulatory network for the periodic genes in *B. distachyon*, we constructed a co-expression gene network based on Pearson’s correlation coefficient, and represented subnetworks at each time point composed of the co-expressed genes. To construct the co-expression gene network, we calculated Pearson’s correlation coefficients (PCC) for all pair-wise combinations of the periodic genes in *B. distachyon.* We found that a GCN contains 3,613 periodic genes linked by 593,826 edges with a PCC ≥ 0.7 for a cutoff threshold of co-expression, and that the number of genes and edges are reduced to 3,369 and 224,473, respectively, with a PCC ≥ 0.8 as its threshold (**Supplementary Figure S3** and Supplementary Table S3). With a color gradient corresponding to the peak time of each gene estimated by our wavelet analysis, we demonstrated that the periodic gene network represents a circular form that is composed of chronologically connected genes (**Figure 3A**). The result is consistent with the diagonal stripe expression patterns (**Figure 2C**). Periodic gene expression is usually regulated by periodically expressed transcription factors. In our list of *Brachypodium* rhythmic genes, we found 192 genes that putatively encode transcription factors (**Figure 3B**). Some of the clock genes encode transcription factors that regulate the periodicity of the expression of many downstream genes in plants by binding to particular *cis*-elements (Romanowski and Yanovsky, 2015; De Caluwé et al., 2016). The comprehensive identification of genes co-expressed with those encoding transcription factors at each peak time, as well as *cis*-motifs in the promoters of rhythmic genes will enable us to infer period-specific transcriptional regulatory networks, which coordinate to particular events in each period. For summarizing gene functions overrepresented in each period, we examined enriched functions of genes based on their peak time. We analyzed peak-time distribution of each of the rhythmically expressed genes and found that 36% of the genes peak at night. Thereafter, assessment of the biological functions of the periodic genes enriched at each peak time revealed that genes putatively involved in various cellular processes, primarily including metabolic processes, are enriched under dark conditions. Moreover, in addition to the processes of genes involved in photosynthesis, those related to responses to stimuli were enriched under daylight conditions, suggesting temporal compartmentalization of biological processes through diurnal changes and providing a landscape of biological events associated with circadian control in the *Brachypodium* transcriptome (**Figure 3C**).

### GRN Inference Based on a Group SCAD Analysis

**FIGURE 4:**
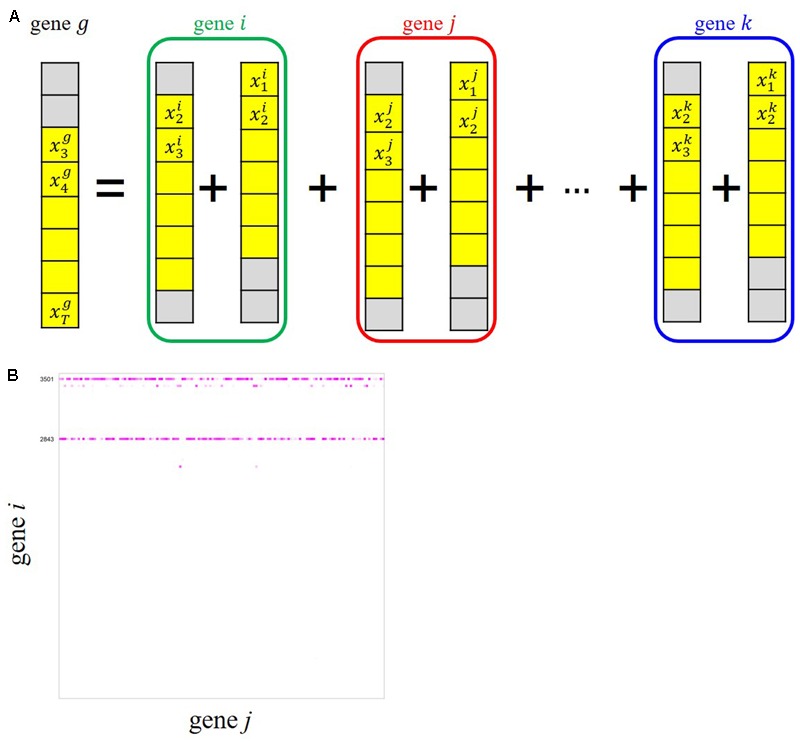
ARX(*p*) (Auto-Regressive eXogenous) model with group smoothly clipped absolute deviation (SCAD) for gene network inference from our time-series transcriptome dataset. **(A)** Schematic representation of an ARX model explaining relations of gene *g* with all the other genes. The colored circles represent grouped variables through *t*–1 and *t–*2 in the case of ARX(2). **(B)** Adjacent matrix *B* of the candidate edges between gene *i* and gene *j* estimated by ARX(3) for 141 isolated genes. The color gradient corresponding to each *B_i,j_* represents the confidence of the gene *i*→ *j* relation in 30 trials, i.e., brightly colored cells in the matrix show reliable relations.

To infer possible dependencies between the rhythmically expressed genes, we applied the ARX(*p*) model with a sparse regularization by group SCAD to the time-series expression dataset of the periodic genes. Here, *p* denotes a time order, i.e., the target variable at time *t* is regressed by the target values observed at *t–1*, …, *t*–*p* as well as exogenous variables observed at the same time points. As schematically shown in **Figure 4A**, the ARX(*p*) model expresses the univariate time-series expression of gene *g* based on a combination of past observations of expressions of genes that are selected from all the rhythmic genes (gene *i*, gene *j*, …, gene *k*), including gene *g* itself, through a variable selection process. We considered three models, ARX(1), ARX(2), and ARX(3), to examine the expression of gene *g* at time *t* from the expression patterns of other genes with time lags 1, 2, and 3, respectively (**Figure 4A**). We randomly produced 30 data matrices with our randomization procedure described in Appendices 1 and 2, and estimated regulatory relations for each of the data matrices. Through our multiple network estimation analysis, we quantitatively assessed regulatory relations for all pair-wise combinations, “gene *i*→ gene *j*,” to infer the candidate directed edges from an adjacent matrix *B* based on *B^m^* (*m* = 1, …, 30) (**Figure 4B**). From the result, we inferred the possible regulatory interactions between the genes, which exceed the confidence threshold set to 20.

**FIGURE 5:**
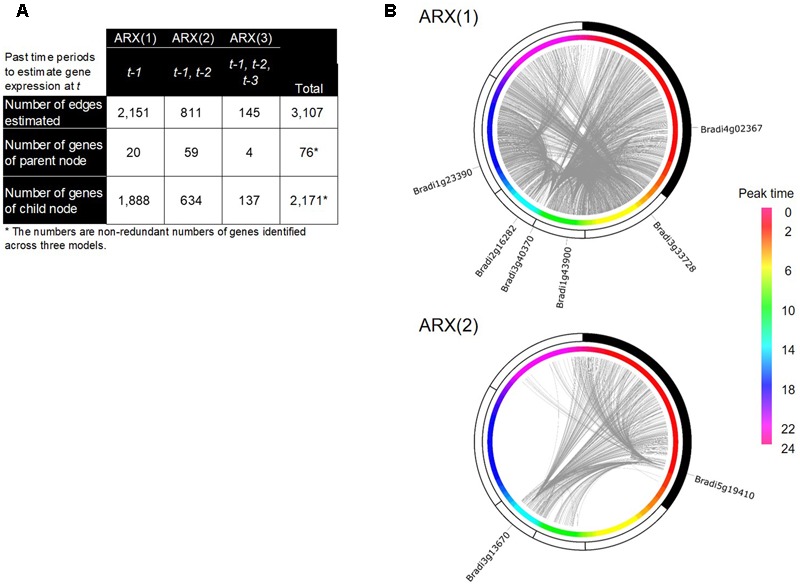
A gene regulatory network (GRN) inferred by the group SCAD method in *B. distachyon*. **(A)** Summarized network structure of the group SCAD networks based on the ARX(1), ARX(2), and ARX(3) models. **(B)** Circular representations of the group SCAD networks with the rhythmic genes and their expression peak time from ARX(1) and ARX(2) models. The nodes of the rhythmic genes are sorted based on their peak time of gene expression. The gray lines represent edges of the networks. Identifiers of genes with more than 50 output edges are shown.

With the inferred network of the *Brachypodium* rhythmic genes using group SCAD (i.e., the group SCAD network), we figured a regulatory network of its leaf transcriptome. This network represented a typical sparse structure of a transcriptional regulatory network, with a small number of parental nodes and a large-number of child nodes (**Figure 5A**), consistent with the findings in several other organisms (Blais and Dynlacht, 2005; Böck et al., 2012; Liu, 2015). We figured a network that contains 3,107 directed edges from 76 to 2,171 genes; in total, 2,187 genes (i.e., 60% of the rhythmically expressed genes). To illustrate the distribution of each of the parent and child nodes along with the time periods of their diurnal expression, we superimposed the group SCAD network onto the estimated peak-time of their expression, and found that several genes of the parent nodes have a remarkable number of edges in particular time periods (**Figure 5B**). The networks inferred by the ARX(1) model were composed of 20 parent nodes and 1,888 child nodes, suggesting that 60% of the expression patterns of the rhythmically expressed genes are explained by the rhythmical expression of 20 genes 4 h earlier. Specifically, each of the six genes of the parent nodes in the ARX(1) model was linked to more than 50 child nodes (Supplementary Table S4), suggesting that these genes could show representative gene expression patterns, which might be spatiotemporal decisive factors for the expression patterns of many genes 4 h later in the *Brachypodium* diurnal transcriptome. Interestingly, we found that the network estimated by the ARX(2) model showed the highest number of genes in parent nodes, indicating that a significant number of possible transcriptional relations exist from 8 h earlier. However, the ARX(3) model showed significantly reduced numbers of such relations between genes. Specifically, we found that Bradi3g13670 and Bradi5g19410 were linked to 220 and 254 child genes, respectively, in the ARX(2) model. The deduced protein sequence of Bradi5g19410 encodes a putative type-2C protein phosphatase (PP2C), which is known to negatively regulate ABA responses and MAPK cascade pathways, thereby playing important roles in stress signal transduction in plants (Hirayama and Umezawa, 2010; Umezawa et al., 2010). A study of genome-wide identification and evolutionary analyses of a putative PP2C gene family recently reported that 86 PP2C genes are present in *B. distachyon* (Cao et al., 2016). Although there is no evidence associated with molecular functions of Bradi5g19410, its rhythmic expression patterns in the diurnal transcriptome and possible spatiotemporal regulatory relations with a number of genes suggest that its crucial roles in the diurnal transcriptome might be related to the circadian gating of physiological responses in *Brachypodium*. The deduced protein sequence of Bradi3g13670 encodes putative beta-carotene isomerase D27, which participates in a pathway leading to the biosynthesis of strigolactones (SLs) (Alder et al., 2012) and functions as endogenous and exogenous signaling molecules in response to various environmental cues (Brewer et al., 2013; Pandey et al., 2016). In addition, previous studies on *FAR-RED ELONGATED HYPOCOTYL3* (*FHY3*), a negative regulator of *RBOH* genes, demonstrated that SL signaling is associated with responses to reactive oxygen species, which is mediated by phytochrome A signaling and the circadian clock, involved in the far-red light response (Lin et al., 2007). Therefore, phytochrome A signaling homeostasis controlled by the orthologous genes should presumably be involved in transcriptional regulation in *Brachypodium* and regulate a number of subordinate genes in its diurnal growth.

## Conclusion

The present study demonstrates the gene networks in the model grass *B. distachyon* on the basis of diurnal transcriptome analyses. Two different approaches, co-expressed gene network and group SCAD network, enabled us to infer the transcriptomic relations of the rhythmic genes. The findings suggested that the combinatorial use of these methods should facilitate the understanding of gene networks that are hierarchically and spatiotemporally organized. Because hierarchical organization and spatiotemporal continuity are the essential features of biological molecular networks, our approach will provide a promising analytical strategy to elucidate a regulatory gene network. Moreover, it will be useful for exploring the candidates of key genes that might influence a number of subordinate genes, according to a series of chronological transcriptome datasets from laboratory and field conditions. These studies are necessary for modeling crop growth associated with agronomic traits in crops.

## Author Contributions

KM and RN conceived, planned, and supervised the project. YO, YU-Y, and MS performed the transcriptome analysis. KT, KI, TY, TS, and KM performed the bioinformatics analysis. SK, HM, HH, SE, and RN performed the statistical analysis. SK, YO, HM, KM, and RN wrote the manuscript.

## Conflict of Interest Statement

The authors declare that the research was conducted in the absence of any commercial or financial relationships that could be construed as a potential conflict of interest.

## Footnotes

1 http://genome.jgi.doe.gov/pages/dynamicOrganismDownload.jsf?organism=Bdistachyon

2 http://genome.jgi.doe.gov/pages/dynamicOrganismDownload.jsf?organism=Athaliana

3 http://genome.jgi.doe.gov/pages/dynamicOrganismDownload.jsf?organism=Osativa

4 http://planttfdb.cbi.pku.edu.cn/index.php?sp=Bdi

5 http://genome.jgi.doe.gov/pages/dynamicOrganismDownload.jsf?organism=Bdistachyon

6 http://bioinf.wehi.edu.au/featureCounts/

7 https://cran.r-project.org/web/packages/WaveletComp/index.html

## Supplementary Material

FIGURE S1 Number of differentially expressed genes (DEGs) between time periods. Numbers in the cells indicate the number of DEGs between the vertical and the horizontal time, each of which corresponds to genes of up- and downregulation, respectively (*q*-value < 1e-5).

FIGURE S2 Results of a correspondence analysis between the putative periodically expressed genes and the sampling time. Dots (orange and red) and rectangles (green and blue) indicate genes and time periods, respectively. Blue rectangles and red dots represent relations between time periods and genes that show the top 100 genes ordered by correspondence contribution.

FIGURE S3 Summary of the co-expressed gene network of the periodic genes in *B. distachyon*. Distributions of number of edges **(A)** and nodes **(B)**, along with the thresholds of Pearson’s correlation coefficient.
