# Supplementary figures and images for: Diurnal Transcriptome and Gene Network Represented through Sparse Modeling in Brachypodium distachyon
Source: Front Plant Sci. 2017 Nov 28;8:2055. doi: 10.3389/fpls.2017.02055 (PMC5712366; doi:10.3389/fpls.2017.02055)

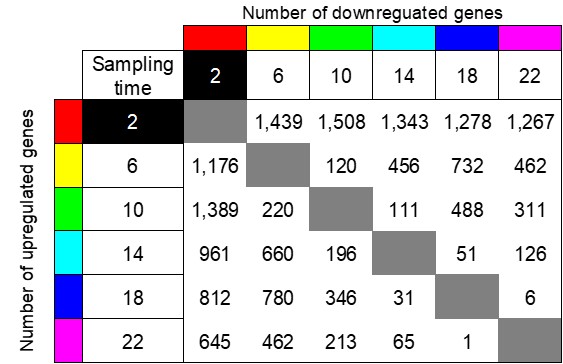

Supplement: FIGURE S1 — Number of differentially expressed genes (DEGs) between time periods. Numbers in the cells indicate the number of DEGs between the vertical and the horizontal time, each of which corresponds to genes of up- and downregulation, respectively (q-value < 1e-5). [file Image_1.JPEG]

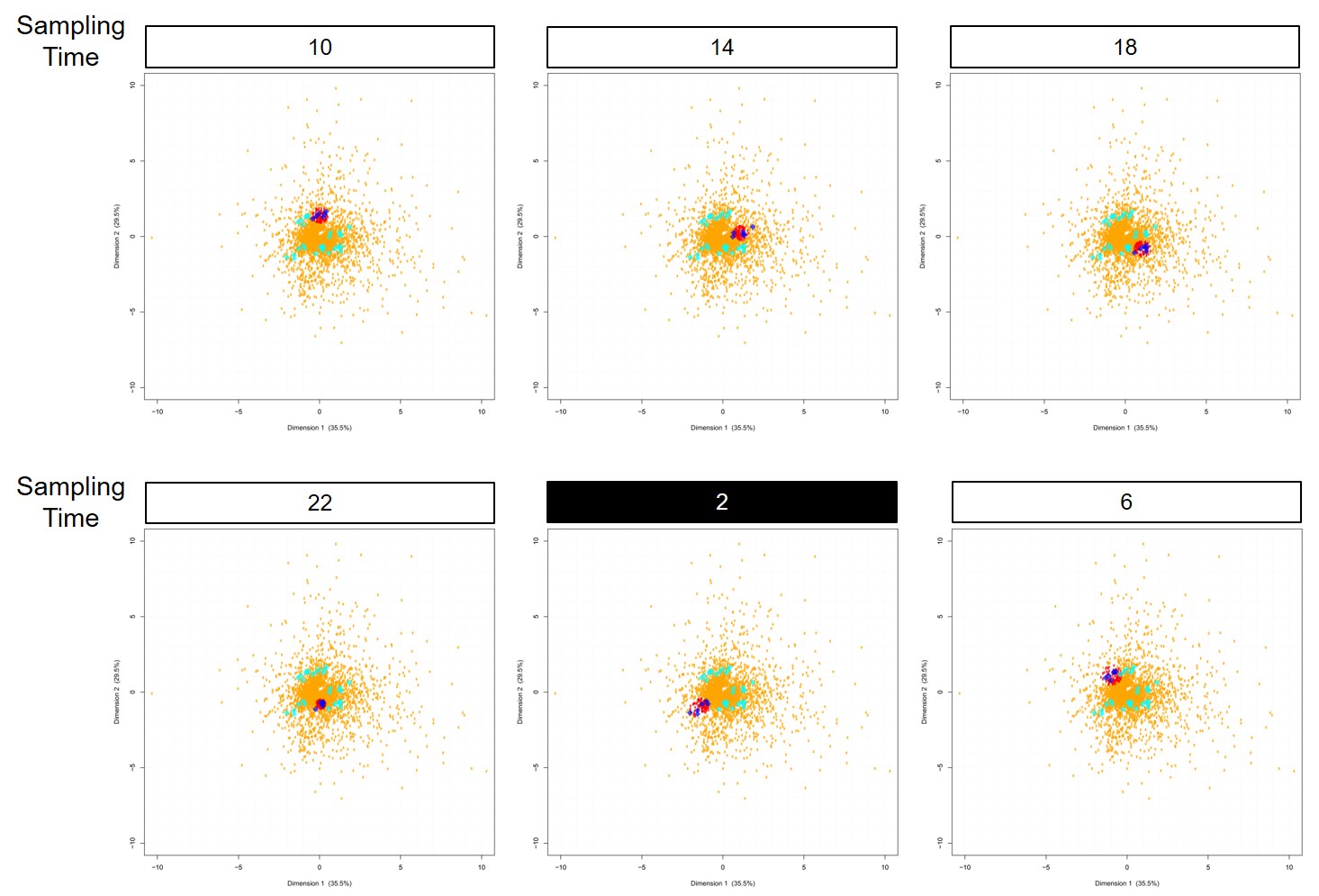

Supplement: FIGURE S2 — Results of a correspondence analysis between the putative periodically expressed genes and the sampling time. Dots (orange and red) and rectangles (green and blue) indicate genes and time periods, respectively. Blue rectangles and red dots represent relations between time periods and genes that show the top 100 genes ordered by correspondence contribution. [file Image_2.JPEG]

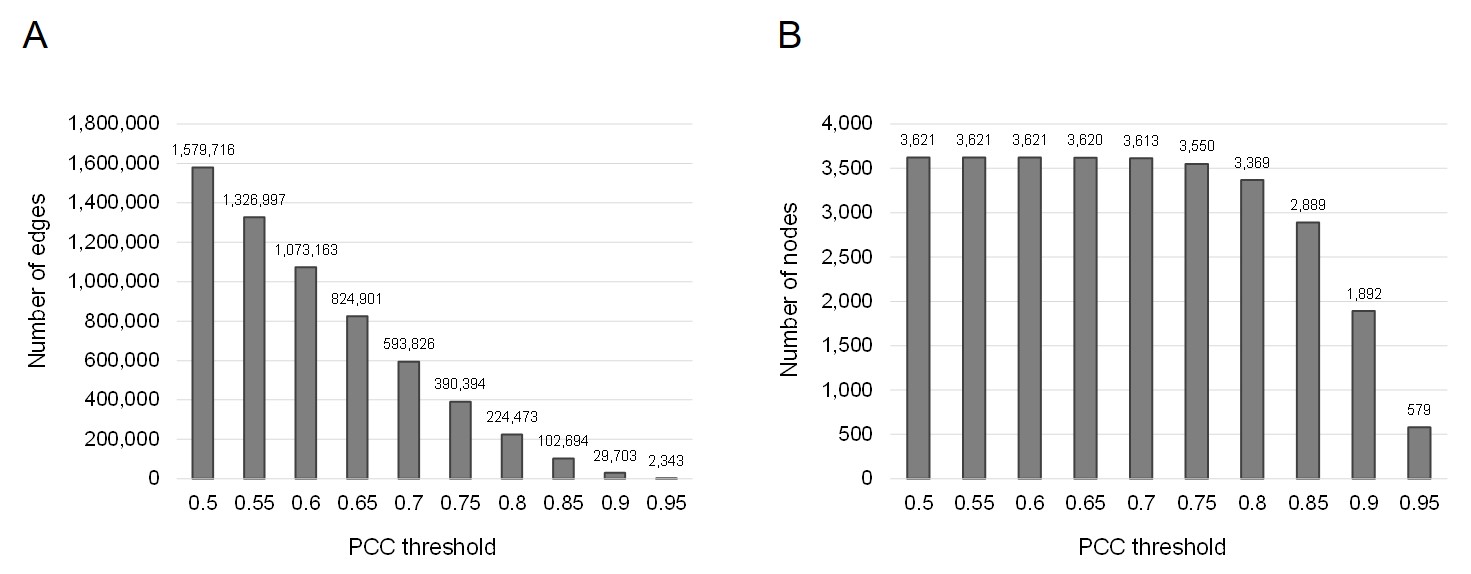

Supplement: FIGURE S3 — Summary of the co-expressed gene network of the periodic genes in B. distachyon. Distributions of number of edges (A) and nodes (B), along with the thresholds of Pearson’s correlation coefficient. [file Image_3.JPEG]
