# Supplementary material for: Diurnal Transcriptome and Gene Network Represented through Sparse Modeling in Brachypodium distachyon
Source: Front Plant Sci. 2017 Nov 28;8:2055. doi: 10.3389/fpls.2017.02055 (PMC5712366; doi:10.3389/fpls.2017.02055)
Supplement: Supplementary file 8 [file Data_Sheet_1.DOCX]

Appendix 1: Details of ARX modeling with group SCAD

We used the group SCAD approach to estimate gene interactions. Suppose that gene expression time series of each gene follows the ARX($p$) model below:

$$x_{t}^{g}=\sum_{i=1}^{N} \sum_{j=1}^{p} \beta_{j}^{i}x_{t-j}^{i}+\varepsilon_{t},$$

where the noise $\varepsilon_{t}$ follows the normal distribution$N(0,\sigma^{2})$. Each coefficient $\beta_{j}^{i}$ shows the interaction from gene $i$ to gene $g$ at order $j$, i.e., $\beta_{j}^{i\to g}$. For the sake of simplicity, we omit the notation $g$ in the coefficient $\beta_{j}^{i}$ by focusing only on a single target gene. Here we define some notations. Let $x_{1:T}^{g}=\left( x_{1}^{g},\ldots,x_{T}^{g} \right)$ be the gene expression time series vector of length $T$. The entire expression matrix $X_{1:T}=\left( x_{1:T}^{1},\ldots,x_{1:T}^{N} \right)$ is of size $T\times N$. We also define $\boldsymbol{\beta}_{j}=\left( \beta_{j}^{1},\ldots,\beta_{j}^{N} \right)^{T}$as a series of coefficients with respect to order $j$, whose element represents the interaction between gene $g$ and gene $i$ $\left( \in\left\{ 1,\ldots,N \right\} \right)$ in order $j$. Then we can formulate the ARX($p$) model as the following matrix form:

$$\boldsymbol{x}_{p+1:T}^{g}=\sum_{j=1}^{p} X_{p+1-j:T-j}\boldsymbol{\beta}_{j}+\boldsymbol{\varepsilon},$$

where $\boldsymbol{\varepsilon}\sim N\left( \boldsymbol{0},\sigma^{2}I_{T-p} \right).$

Although we can estimate the weight parameter $\boldsymbol{\beta}_{j}$ with the least squares estimator, we used the sparse estimation method based on the SCAD penalty, because gene networks are normally sparse. The penalty term is defined as

$$p_{\lambda}\left( \beta\right)=\left\{ \begin{aligned} \lambda\left| \beta\right| \mathrm{if}0\leq\left| \beta\right|\leq\lambda, \\ -\frac{\beta^{2}-2a\lambda\left| \beta\right|+\lambda^{2}}{2\left( a-1 \right)}\mathrm{if}\lambda\leq\left| \beta\right|\leq a\lambda, \\ \frac{\left( a+1 \right)\lambda^{2}}{2} otherwise, \end{aligned} \right.$$

where $a$ and $\lambda$ are positive constants. The penalty can make irrelevant weights shrink exactly to zero, i.e., we can identify interactive genes related to gene $g$. Following the literature (Michailidis and D’Alche-Buc, 2013), we fixed $a=3.7$ and estimated the tuning parameter $\lambda$ by cross validation.

Moreover, we incorporate the estimation with group effects to determine the interactions without depending on a specific time lag. With the upper index, let $\boldsymbol{\beta}^{i}=\left( \beta_{1}^{i},\ldots,\beta_{p}^{i} \right)^{T}$. The group SCAD estimator can be obtained as the result of the following optimization regarding all the coefficients $\boldsymbol{\beta}$:

$$\underset{\boldsymbol{\beta}}{\mathrm{argmin}} \left\{ \left\| \boldsymbol{x}_{p+1:T}^{g}-\sum_{j=1}^{p} X_{p+1-j:T-j}\boldsymbol{\beta}_{j} \right\|_{2}^{2}+\sum_{i=1}^{N} P_{\lambda}\left( \left\| \boldsymbol{\beta}^{i} \right\|_{2} \right) \right\},$$

where $\left\| \boldsymbol{\beta}^{i} \right\|_{2}$ indicates the $l_{2}$ norm of $\boldsymbol{\beta}^{i}$. When $\boldsymbol{\beta}^{i}\boldsymbol{\neq}\boldsymbol{0}$, we conclude that gene $i$ regulates gene $g$. In contrast, when they do not hold any interactions, all the elements of $\boldsymbol{\beta}^{i}$ become zero, because of the group effect. Applying the above procedures to every gene, we can get the adjacent matrix $B$, whose element $B_{i,j}$ takes one only if gene $i$ regulates gene $j$. The actual analysis was done by grppenalty package in R (https://cran.r-project.org/web/packages/grppenalty/index.html).
