# Supplementary material for: Diurnal Transcriptome and Gene Network Represented through Sparse Modeling in Brachypodium distachyon
Source: Front Plant Sci. 2017 Nov 28;8:2055. doi: 10.3389/fpls.2017.02055 (PMC5712366; doi:10.3389/fpls.2017.02055)
Supplement: Supplementary file 9 [file Data_Sheet_2.DOCX]

Appendix 2: Randomization

To cope with noise in our data, we artificially created plural datasets with permutation based on the diurnal transcriptome dataset. In the transcriptome dataset, every gene virtually has expression time series measured at 36 time points. Its values are based on 12 time points across two days with three biological replicates. This conjunction results in configuring a virtual $N\times36$ data matrix. Since we found most identical gene expression patterns between the two days, each time series can be viewed as the one measured for six replicates. We permuted the six observations for individual genes while maintaining the time order of each of the six replicates, so as not to break their temporal structure, which results in the configuration of another data matrix. We created 30 sets of virtual $N\times36$ data matrices. Subsequently, we estimated their adjacent matrixes $B^{1},\ldots,B^{30}$ by applying group SCAD to every data set. As our final outcome, we extracted the gene interactions that satisfy $B_{i,j}\geq20$, where $B=\sum_{m} B^{m}$.
